# Health Consciousness and Dietary Behavior: A Theory of Planned Behavior Analysis of Organic Food Adoption Among Young Consumers

**DOI:** 10.3390/bs16061006

**Published:** 2026-06-16

**Authors:** Aracelly Núñez-Naranjo, Diana Morales-Urrutia, Luis Mantilla-Falcón, Oscar Ibarra-Torres, Patricio Córdova

**Affiliations:** 1 Centro de Investigación en Ciencias Humanas y de la Educación-CICHE, Facultad de Ciencias de la Educación, Universidad Tecnológica Indoamérica, Ambato 180103, Ecuador; 2 Facultad de Ciencias Administrativas, Universidad Técnica de Ambato, Grupo de Investigación DeTEI, Ambato 180206, Ecuador; dc.moralesu@uta.edu.ec; 3 Facultad de Contabilidad y Auditoría, Universidad Técnica de Ambato, Ambato 180206, Ecuador; luismmantilla@uta.edu.ec; 4 Facultad de Ingeniería en Sistemas, Electrónica e Industrial, Universidad Técnica de Ambato, Ambato 180206, Ecuador; of.ibarra@uta.edu.ec (O.I.-T.); edgarpcordovac@uta.edu.ec (P.C.)

**Keywords:** health consciousness, dietary health behavior, theory of planned behavior, behavioral intention, perceived behavioral control, organic food consumption, preventive health behavior, emerging markets

## Abstract

The adoption of healthier dietary behaviors has become a critical public health concern, particularly among young populations facing structural and economic constraints. Within this context, organic food consumption can be understood not only as a market choice but as a form of health-related behavior influenced by psychological factors. Drawing on the Theory of Planned Behavior, this study examines how health consciousness and core cognitive determinants shape dietary health behavior through their influence on behavioral intention and self-reported consumption patterns. A cross-sectional quantitative design was employed using data from 384 young consumers in an emerging market context (Ambato, Ecuador). The proposed model was tested using covariance-based structural equation modeling (CB-SEM). The findings indicate that perceived behavioral control is the strongest predictor of intention to engage in organic food consumption, followed by attitude and subjective norms. Health consciousness is positively associated with attitude and indirectly influences behavioral intention through this pathway. No significant relationship was found between perceived behavioral control and attitude. Behavioral intention shows a strong association with self-reported consumption behavior. These results highlight the central role of perceived feasibility in shaping health-related dietary behaviors in constrained contexts, where structural barriers may limit the translation of positive attitudes into action. The study contributes to the health psychology literature by providing context-sensitive evidence on how cognitive and motivational factors interact within the TPB framework to influence dietary behavior. Implications for promoting healthier consumption patterns emphasize the need to address both psychological drivers and structural constraints.

## 1. Introduction

In recent years, the promotion of healthier dietary behaviors has become a central priority in public health, given their role in preventing chronic diseases and improving overall well-being (Andersen et al., 2022; Bryła, 2016). Among these behaviors, the consumption of organic food has gained increasing attention, as it is often associated with perceived health benefits, reduced exposure to harmful substances, and alignment with healthier lifestyles (FAO, 2007; Kim & Lee, 2023; Sandri et al., 2025). Recent empirical studies have further emphasized the growing importance of dietary behavior as a key determinant of health outcomes, particularly in relation to food choice and preventive health practices (J. Wang et al., 2023; Yadav & Pathak, 2016).

Although organic food consumption is frequently examined from a consumer behavior perspective, it can also be conceptualized as a form of health-related behavior shaped by psychological, cognitive, and contextual factors (Carrión-Bósquez et al., 2024; Nguyen et al., 2023). In this sense, understanding why individuals adopt—or fail to adopt—healthier dietary choices requires moving beyond market-based explanations and focusing on the underlying motivational and cognitive processes that drive behavior (Verplanken & Orbell, 2022). This perspective is consistent with recent research, where dietary choices are increasingly framed as health-related behaviors influenced by psychosocial and environmental determinants.

The Theory of Planned Behavior (TPB) provides a well-established framework for explaining health-related behaviors, including dietary decisions (Ajzen, 1991; Fishbein & Ajzen, 1974). According to TPB, behavior is primarily determined by behavioral intention, which is influenced by attitude, subjective norms, and perceived behavioral control. While this framework has been widely applied, evidence suggests that the relative importance of these components may vary depending on contextual constraints, particularly in emerging markets where access, affordability, and availability limit behavioral feasibility (Carrión-Bósquez et al., 2023; Weissmann & Hock, 2022). Recent empirical studies have applied the TPB to explain health-related and sustainable consumption behaviors, confirming its robustness while highlighting the importance of contextual and psychological factors in shaping dietary decision-making (Aschemann-Witzel & Zielke, 2017; Nguyen et al., 2023; Tandon et al., 2020; Vermeir & Verbeke, 2006).

In parallel, health consciousness has been identified as a key psychological factor influencing dietary behavior (Nagaraj, 2021; Nguyen et al., 2023). Individuals with higher levels of health consciousness are more likely to engage in behaviors aimed at maintaining or improving their health (C.-Y. Wang & Tsai, 2025; Widyasari & Haryanto, 2013). However, prior research has predominantly examined health consciousness as a direct predictor of behavioral intention, overlooking its broader role in shaping the cognitive structure underlying health-related decision-making (Harjadi & Gunardi, 2022). J. Wang et al. (2020) have also highlighted that health concerns are a key determinant of preventive health habits and dietary choices, particularly in the context of organic and sustainable consumption Against this background, this study examines how health consciousness operates within the TPB framework to influence dietary health behavior, specifically in the context of organic food consumption among young consumers in an emerging market characterized by structural constraints.

### 1.1. Literature Review

#### 1.1.1. Theory of Planned Behavior and Organic Food Consumption

The Theory of Planned Behavior originally proposed by (Ajzen, 1991), is one of the most widely used frameworks for explaining deliberate human behavior across social and health-related contexts. According to the TPB, behavior is primarily determined by behavioral intention, which is shaped by three core cognitive components: attitude, subjective norms, and perceived behavioral control (Fishbein & Ajzen, 2011). Attitude reflects the individual’s evaluation of the behavior, subjective norms capture perceived social expectations, and perceived behavioral control represents the perceived feasibility of performing the behavior given available resources and constraints.

The TPB has been extensively applied to explain organic food consumption and related dietary behaviors, with consistent evidence showing that behavioral intention is a key predictor of actual behavior (Ahmed et al., 2021; Loera et al., 2022). Recent studies have further confirmed the applicability of the TPB in explaining health-related and sustainable food consumption, highlighting its robustness across different cultural and economic contexts (Tandon et al., 2020; Yadav & Pathak, 2016). These studies emphasize that the TPB provides a flexible framework capable of capturing both individual motivations and contextual influences on dietary decision-making.

However, empirical findings are not entirely consistent regarding the relative importance of TPB antecedents. While several studies identify attitude as the strongest predictor of behavioral intention toward organic food consumption, others report that perceived behavioral control exerts a greater influence, particularly in contexts characterized by economic and accessibility constraints. These contrasting findings suggest that the predictive power of TPB components may depend on contextual conditions rather than representing universal behavioral mechanisms. In developed markets, where organic products are more accessible, positive evaluations may be sufficient to stimulate intention. In contrast, in emerging markets, feasibility considerations often become more salient because consumers must evaluate whether healthy consumption choices are realistically attainable. This inconsistency highlights the need to further investigate how TPB components operate under different contextual conditions (Carrión-Bósquez et al., 2023; Tandon et al., 2020; Weissmann & Hock, 2022). In line with this perspective, (Bryła, 2016; Kushwah et al., 2019) has shown that perceived behavioral control becomes especially relevant in constrained environments, where structural barriers limit individuals’ ability to translate positive attitudes into actual behavior. 

Subjective norms, in contrast, often exhibit weaker or context-dependent effects, particularly when consumption decisions are perceived as personal or health-related rather than socially driven. This pattern is consistent with findings in health behavior research, where individual cognitive evaluations and perceived control tend to outweigh social influence in shaping behavioral intentions.

These inconsistencies suggest that TPB components do not operate uniformly across contexts. In emerging markets, structural constraints—such as limited availability, high prices, and institutional uncertainty—may alter the relative influence of cognitive predictors. Under such conditions, perceived behavioral control may become more salient, as it captures the extent to which individuals perceive organic food consumption as feasible rather than merely desirable.

Therefore, rather than questioning the validity of the TPB, recent research points to the need to better understand how its internal configuration varies across contexts and how additional psychological and contextual factors may shape its cognitive components (Michaelidou & Hassan, 2008; Yadav & Pathak, 2016; Prakash et al., 2018). From this perspective, perceived behavioral control may also influence individuals’ evaluative judgments regarding organic food consumption. When consumers perceive a behavior as feasible and manageable, they may develop more favorable evaluations toward that behavior. Similarly, social influences reflected through subjective norms may contribute to shaping positive evaluations when healthy consumption practices are socially encouraged and reinforced. These relationships have been explored in previous consumer behavior studies that recognize interactions among cognitive antecedents rather than treating them exclusively as independent predictors.

Although the TPB traditionally considers these antecedents as distinct predictors, previous research has shown that their relationships may vary across contexts and may involve indirect pathways that contribute to intention formation. Furthermore, perceived behavioral control may also influence attitude formation. When individuals perceive that performing a behavior is feasible and within their control, they are more likely to develop favorable evaluations toward that behavior. Accordingly, a positive relationship between perceived behavioral control and attitude toward organic food consumption is expected.

#### 1.1.2. Health Consciousness Within the TPB Framework

Health consciousness has been consistently identified as a relevant determinant of food-related decision-making, particularly in the context of organic products and dietary health behavior (Nagaraj, 2021; Nguyen et al., 2023; Parashar et al., 2023). It refers to the degree to which individuals are aware of, concerned about, and motivated to improve their health through consumption choices. Empirical studies show that individuals with higher levels of health consciousness tend to develop more favorable attitudes toward organic foods and, in many cases, stronger behavioral intentions (C.-Y. Wang & Tsai, 2025; Widyasari & Haryanto, 2013). Consistent with prior research, health consciousness has been recognized as an important psychological antecedent of food-related decision-making, particularly regarding the consumption of organic and functional food products (Bryła, 2016; J. Wang et al., 2020).

Although previous studies consistently recognize health consciousness as an important determinant of food-related behavior, important differences remain regarding its role within behavioral decision-making processes. Some studies conceptualize health consciousness as a direct predictor of behavioral intention, suggesting that greater concern for health automatically increases the likelihood of engaging in healthy consumption practices. Other studies argue that its influence is more indirect, operating through cognitive evaluations such as attitudes and perceptions regarding food-related choices. These differing perspectives indicate that the mechanisms through which health consciousness influences behavior remain insufficiently understood. Consequently, further research is needed to determine whether health consciousness acts primarily as a motivational driver or as a broader cognitive factor shaping TPB components (Ajzen, 2020; Conner & Armitage, 1998; Sniehotta et al., 2014).

These considerations suggest that health consciousness may operate not only as a direct predictor but also as a distal cognitive factor that shapes individuals’ evaluations of health-related consumption choices. In the context of the present study, health consciousness is specifically examined through its relationship with attitude toward organic food consumption, which represents a key cognitive antecedent within the TPB framework. In this sense, rather than acting solely at the level of behavioral intention, health consciousness may function as a broader cognitive factor that shapes how individuals evaluate health-related consumption choices. However, in the present study, health consciousness is examined exclusively through its influence on attitude toward organic food consumption. This conceptualization aligns with emerging perspectives in behavioral science that emphasize the interaction between motivational and cognitive determinants in shaping health-related behavior.

Despite growing evidence regarding the relationship between health consciousness and food-related decision-making, empirical studies examining its role within the TPB framework remain limited, particularly in emerging market contexts. More specifically, little attention has been given to the extent to which health consciousness shapes attitude toward organic food consumption and its subsequent influence on behavioral intention and dietary behavior.

This gap is particularly relevant, as failing to account for this broader cognitive role may lead to an incomplete understanding of how health-related motivations influence dietary behavior under conditions of structural constraint. Addressing this gap is essential for advancing both theoretical and empirical research on health-related behavior in real-world settings.

The proposed relationships are grounded in the assumption that individuals with higher levels of health consciousness tend to process food-related information more carefully and develop stronger evaluations regarding behaviors perceived as beneficial to their health. Consequently, health consciousness is expected to contribute to the formation of favorable attitudes toward organic food consumption, which subsequently influence behavioral intention and dietary behavior.

#### 1.1.3. Intention–Behavior Gap and Contextual Constraints in Latin America

A well-documented phenomenon in research on sustainable and health-related consumption is the gap between behavioral intention and actual behavior (Macas-Quito et al., 2022; Schäufele & Janssen, 2021). Individuals often express favorable attitudes and strong intentions toward organic food consumption; however, these do not always translate into consistent dietary behavior. This discrepancy underscores the importance of considering contextual factors and perceived barriers that may hinder the translation of behavioral intentions into actual behavior. Previous studies have similarly documented the persistence of the intention–behavior gap in health-related and sustainable consumption contexts.

In Latin American markets, structural factors such as high prices, limited product availability, and low trust in certification systems play a significant role in shaping consumption outcomes (Carrión-Bósquez et al., 2023; Eyinade et al., 2021). Under these conditions, perceived behavioral control becomes a key mechanism linking behavioral intention to dietary behavior, as it reflects individuals’ perceptions of affordability, accessibility, and practical feasibility (La Barbera & Ajzen, 2021; Vicente et al., 2021). When perceived control is limited, even strong behavioral intentions may fail to result in actual behavior. This pattern has also been documented in prior studies conducted in Latin American contexts, where structural constraints significantly reduce the likelihood that behavioral intentions are converted into consistent dietary behavior (Kushwah et al., 2019).

While prior studies in the region have confirmed the relevance of TPB predictors, less attention has been given to how these variables interact through indirect pathways. In particular, the potential mediating roles of attitude and behavioral intention in linking contextual and cognitive factors to dietary behavior have not been consistently examined. This limitation restricts a more nuanced understanding of how internal motivations and external constraints jointly shape behavioral outcomes, especially in constrained environments.

Building on this perspective, the present study adopts an integrative approach that examines both direct and indirect relationships within the TPB framework. Specifically, it considers health consciousness as a distal cognitive factor influencing TPB components, while also allowing for mediated relationships among attitude, perceived behavioral control, behavioral intention, and self-reported dietary behavior. This approach responds to recent calls in the literature to better understand the interplay between psychological and contextual determinants of health-related behavior.

Taken together, the literature suggests that dietary behavior related to organic food consumption among young consumers is shaped by the interaction of health-related motivations, cognitive evaluations, social influences, and perceptions regarding behavioral feasibility. Within this framework, health consciousness contributes to the development of favorable evaluations toward organic food consumption, while subjective norms and perceived behavioral control influence the formation of behavioral intentions through complementary cognitive processes. Although the TPB traditionally considers these antecedents as distinct predictors, previous research has shown that their relationships may vary across contexts and may involve indirect pathways that contribute to intention formation. Based on these theoretical arguments, the following hypotheses are formulated.

Beyond addressing the identified research gaps, this study contributes to both the consumer behavior literature and the Theory of Planned Behavior literature in three ways. First, it extends the application of the TPB by examining health consciousness as a distal cognitive factor associated with organic food consumption. Second, it simultaneously evaluates both direct and indirect relationships among TPB components through a structural equation modeling framework. Third, it provides empirical evidence from Ecuador, an emerging-market context that remains underrepresented in the literature on organic food consumption and health-related dietary behavior.

**H1.**  *Health consciousness is positively associated with attitude toward organic products*.

**H2.**  *Subjective norms are positively associated with attitude toward organic products*.

**H3.**  *Perceived behavioral control is positively associated with attitude toward organic products*.

**H4.**  *Perceived behavioral control is positively associated with subjective norms*.

**H5.**  *Attitude toward organic products is positively associated with behavioral intention*.

**H6.**  *Subjective norms are positively associated with behavioral intention*.

**H7.**  *Perceived behavioral control is positively associated with behavioral intention*.

**H8.**  *Behavioral intention is positively associated with self-reported dietary behavior*.

**H9.**  *The relationship between subjective norms and behavioral intention is partially mediated by attitude*.

**H10.**  *The relationship between perceived behavioral control and behavioral intention is partially mediated by attitude*.

**H11.**  *The relationship between perceived behavioral control and dietary behavior is partially mediated by behavioral intention*.

**H12.**  *Health consciousness is indirectly associated with behavioral intention through its relationship with attitude*.

## 2. Materials and Methods

### 2.1. Data Collection and Participants

This study employed a quantitative cross-sectional design with an explanatory purpose to examine the relationships among psychological factors associated with behavioral intention and self-reported dietary behavior related to organic food consumption among young consumers. Given the confirmatory nature of the research and its theoretical grounding in the Theory of Planned Behavior, a covariance-based Structural Equation Modeling (CB-SEM) approach was employed. CB-SEM is particularly appropriate for theory testing and model evaluation because it allows the simultaneous estimation of measurement and structural components while providing a comprehensive assessment of overall model fit.

The target population consisted of young consumers residing in the city of Ambato, Ecuador. Young consumers were defined as individuals aged between 18 and 35 years, consistent with previous studies on sustainable consumption and health-related food choices. Participants were recruited through a non-probability sampling approach based on voluntary participation. The questionnaire was distributed digitally among young consumers residing in Ambato who met the established age criteria. Although the sampling procedure does not allow statistical representativeness of the target population, it is consistent with previous consumer behavior studies employing Structural Equation Modeling to examine theoretical relationships among latent constructs. The final sample comprised 384 valid responses, exceeding the minimum thresholds commonly recommended for CB-SEM analysis.

The sample was composed of young consumers with heterogeneous socioeconomic backgrounds. The inclusion of individuals within the 18–35 age range allowed us to capture variability in purchasing capacity, exposure to organic products, and consumption habits.

Data was collected through a structured self-administered questionnaire distributed in digital format. Participation was voluntary and anonymous, and informed consent was obtained from all respondents prior to participation. The study adhered to ethical standards applicable to survey-based social science research and complied with institutional ethical guidelines governing questionnaire-based research.

### 2.2. Questionnaire Design

The data collection instrument consisted of a questionnaire comprising 21 measurement items grouped into six latent constructs derived from the TPB framework and the inclusion of health consciousness:

- Attitude toward organic products (ACT)
- Subjective Norms (NP)
- Perceived Behavioral Control (CCP)
- Health Consciousness (CS)
- Behavioral Intention (INT)
- Self-reported Dietary Behavior (COMP)

All items were measured using a five-point Likert scale ranging from 1 (strongly disagree) to 5 (strongly agree). Measurement items were adapted from previously validated scales widely used in studies on organic food consumption and pro-environmental behavior. Minor semantic adjustments were made to ensure contextual relevance to the Ecuadorian setting without altering the conceptual meaning of the original scales.

Importantly, the construct self-reported dietary behavior captures respondents’ reported frequency and consistency of organic food consumption as part of their dietary practices.

### 2.3. SEM Assumptions and Analytical Procedure

Prior to model estimation, the dataset was screened for completeness and consistency. The analysis followed the two-step approach commonly recommended in covariance-based structural equation modeling (CB-SEM), consisting of the evaluation of the measurement model followed by the assessment of the structural model.

Because CB-SEM with maximum likelihood estimation assumes approximate multivariate normality, univariate normality was examined prior to model estimation. Skewness and kurtosis values were calculated for all observed variables. The obtained values remained within acceptable thresholds (|skewness| < 2; |kurtosis| < 7), supporting the use of maximum likelihood estimation.

Confirmatory Factor Analysis (CFA) and Structural Equation Modeling (SEM) were performed using AMOS 29 Graphics software with maximum likelihood estimation. Following the recommendations of Anderson and Gerbing (1988), the measurement model was evaluated before estimating the structural relationships among latent constructs.

To assess the potential presence of common method bias (CMB), Harman’s single-factor test was conducted. All measurement items were entered into an unrotated exploratory factor analysis. The first factor accounted for less than 50% of the total variance, indicating that common method variance was not likely to represent a serious threat to the validity of the results. This procedure is widely used as a diagnostic assessment in survey-based behavioral research employing self-reported measures.

### 2.4. Measurement Model Evaluation Criteria

Prior to the assessment of the measurement model, Harman’s single-factor test was performed to evaluate the potential presence of common method bias. The first unrotated factor explained 38.7% of the total variance, which is below the commonly accepted threshold of 50%. Therefore, common method bias was not considered a significant concern in this study.

The measurement model was evaluated using Confirmatory Factor Analysis (CFA). Reliability was assessed through Cronbach’s alpha and Composite Reliability (CR). Convergent validity was examined through standardized factor loadings and Average Variance Extracted (AVE).

Following commonly accepted recommendations, standardized factor loadings above 0.50, CR values above 0.70, AVE values above 0.50, and Cronbach’s alpha coefficients above 0.70 were considered indicative of acceptable reliability and convergent validity.

Discriminant validity was assessed using both the Fornell–Larcker criterion and the Heterotrait–Monotrait ratio (HTMT).

### 2.5. Structural Model Evaluation Criteria

The structural model was evaluated through multiple goodness-of-fit indicators, including chi-square divided by degrees of freedom (χ^2^/df), Comparative Fit Index (CFI), Incremental Fit Index (IFI), Tucker–Lewis Index (TLI), Normed Fit Index (NFI), Root Mean Square Error of Approximation (RMSEA), and Standardized Root Mean Square Residual (SRMR).

Following established SEM guidelines, χ^2^/df values below 5.0, CFI, IFI, TLI, and NFI values approaching or exceeding 0.90, RMSEA values below 0.08–0.10, and SRMR values below 0.08 were considered indicative of acceptable model fit.

### 2.6. Robustness Considerations

Given the cross-sectional design and the use of self-reported measures, results should be interpreted with caution. The relationship between Behavioral intention and self-reported purchasing behavior may be influenced by common method bias and does not capture longitudinal behavioral patterns. Future research could incorporate longitudinal designs, objective behavioral data, or experimental approaches to strengthen causal inference and reduce potential bias.

## 3. Results

### 3.1. Sample Description

In order to characterize the study participants, Table 1 presents the main socio-demographic characteristics of the sample. The respondents consisted of young consumers aged between 18 and 35 years residing in Ambato, Ecuador. The sample included individuals from diverse socioeconomic backgrounds, providing variability in purchasing capacity and exposure to organic food products.

Table 1 presents the socio-demographic characteristics of the respondents. The sample consisted of 384 young consumers residing in Ambato, Ecuador. Female respondents represented 54.2% of the sample, while males accounted for 45.8%. The largest age group was between 18 and 24 years (41.1%), followed by participants aged 25–29 years (32.3%). Most respondents reported having undergraduate-level education (64.3%), and more than half were employed (51.6%). Regarding income, the largest proportion of participants reported monthly earnings between USD 461 and USD 800 (38.8%). These demographic characteristics are consistent with previous studies on organic food consumption, which have found that younger, more educated consumers tend to exhibit greater interest in health-related and sustainable food choices (Kushwah et al., 2019; Prakash et al., 2018; Yadav & Pathak, 2016). Furthermore, employment status and moderate income levels have been associated with greater exposure to and awareness of organic products, although economic constraints may still influence purchasing decisions (Scalco et al., 2017). Overall, the sample reflects a heterogeneous group of young consumers with varying educational, occupational, and income backgrounds, providing an appropriate context for examining organic food consumption behavior.

### 3.2. Measurement Model Assessment

The measurement model was evaluated through Confirmatory Factor Analysis (CFA) (Table 2). Standardized factor loadings, reliability indicators, and validity measures (Appendix A) were examined to assess the adequacy of the latent constructs.

The results indicate that most standardized factor loadings exceeded the recommended threshold of 0.60. A limited number of indicators showed loadings slightly below this value but were retained due to their theoretical relevance and acceptable contribution to construct validity.

In addition to factor loadings, construct reliability and validity were assessed using Cronbach’s alpha, Composite Reliability (CR), Average Variance Extracted (AVE), the Fornell–Larcker criterion, and the Heterotrait–Monotrait ratio (HTMT). As reported in Appendix A, all constructs exceeded the recommended thresholds for internal consistency reliability (Cronbach’s α > 0.70; CR > 0.70) and convergent validity (AVE > 0.50). Furthermore, the Fornell–Larcker criterion and HTMT values supported discriminant validity, indicating that the latent constructs are empirically distinct and adequately measured.

### 3.3. Model Fit

The overall fit of the structural model was assessed using multiple goodness-of-fit indices (Table 3). Model fit was evaluated through the joint interpretation of χ^2^/df = 3.76, CFI = 0.906, IFI = 0.907, TLI = 0.891, NFI = 0.877, RMSEA = 0.084, and SRMR = 0.061. While CFI, IFI, and SRMR met commonly recommended thresholds, TLI and NFI were slightly below the conventional criterion of 0.90, and RMSEA exceeded more conservative cut-off values. Therefore, the model fit should be interpreted cautiously as providing moderate support for the proposed theoretical specification rather than indicating an optimal fit. Following established SEM guidelines, model adequacy was assessed through the combined interpretation of multiple fit indices rather than reliance on a single statistic (Hair et al., 2019).

### 3.4. Structural Model Results

The estimated relationships among latent constructs are presented in Table 4.

The results indicate that perceived behavioral control has a strong and statistically significant effect on subjective norms (β = 0.829, *p* < 0.001). Health consciousness is also strongly associated with attitude toward organic food (β = 0.873, *p* < 0.001), while subjective norms show a moderate but significant effect on attitude (β = 0.296, *p* < 0.001). In contrast, the relationship between perceived behavioral control and attitude is not statistically significant (β = 0.009, *p* = 0.921).

Regarding behavioral intention, perceived behavioral control emerges as the strongest predictor (β = 0.750, *p* < 0.001), followed by attitude (β = 0.324, *p* < 0.001) and subjective norms (β = 0.170, *p* = 0.015). Finally, behavioral intention shows a strong and significant association with self-reported dietary behavior (β = 0.867, *p* < 0.001).

### 3.5. Indirect Effects

To assess the significance of the proposed indirect relationships, bootstrap procedures with 5000 resamples and bias-corrected 95% confidence intervals were conducted. The results are presented in Table 5.

The bootstrap analysis revealed that the indirect effect of subjective norms on behavioral intention through attitude was statistically significant (β = 0.096, 95% CI [0.041, 0.151]), providing support for H9. Similarly, health consciousness exhibited a significant indirect effect on behavioral intention through attitude (β = 0.283, 95% CI [0.191, 0.377]), supporting H12.

In addition, perceived behavioral control showed a significant indirect effect on dietary behavior through behavioral intention (β = 0.650, 95% CI [0.541, 0.758]), supporting H11 and highlighting the central role of intention in the translation of cognitive evaluations into behavior.

However, the indirect effect of perceived behavioral control on behavioral intention through attitude was not statistically significant (β = 0.003, 95% CI [−0.014, 0.021]), as the confidence interval included zero. Therefore, H10 was not supported.

These findings provide additional evidence regarding the indirect mechanisms through which cognitive and motivational factors contribute to dietary behavior related to organic food consumption.

### 3.6. Hypothesis Testing Summary

To facilitate interpretation of the empirical findings, Table 6 summarizes the results of hypothesis testing by indicating whether each proposed hypothesis was supported or not supported. The decision was based on the statistical significance and direction of the estimated direct and indirect effects.

As shown in Table 4, ten of the twelve proposed hypotheses were supported. The only hypotheses not supported were H3 and H10. Specifically, perceived behavioral control was not significantly associated with attitude toward organic products, and its indirect effect on behavioral intention through attitude was also not significant. These findings suggest that perceived behavioral control operates primarily through its direct effect on behavioral intention and through its indirect effect on dietary behavior via intention, rather than through attitudinal evaluations.

**Figure 1:**
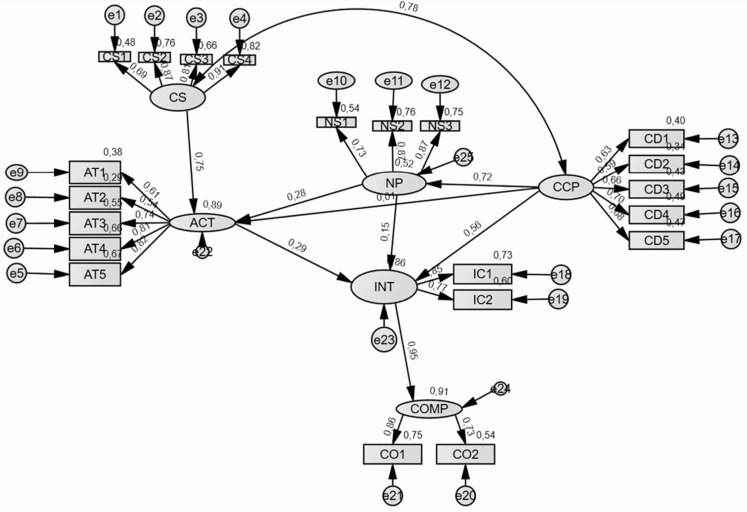
Structural equation model.

To facilitate interpretation of the direct and indirect relationships identified in the model, Figure 1 presents the standardized estimates of the final structural model, including the structural paths among latent constructs and their associated factor loadings.

## 4. Discussion

The results of the study provide empirical evidence on the relationships among health consciousness, the core components of the Theory of Planned Behavior, behavioral intention, and self-reported dietary behavior in the context of organic food consumption among young individuals in an emerging market. From a health psychology perspective, these findings contribute to a more nuanced understanding of how cognitive, motivational, and contextual mechanisms jointly shape the adoption of healthier dietary behaviors under structural constraints.

Overall, the findings are consistent with prior TPB-based research, confirming that attitude, subjective norms, and perceived behavioral control are positively associated with behavioral intention, and that intention is strongly associated with self-reported dietary behavior (Ajzen, 1991; Fishbein & Ajzen, 2011). This reinforces the robustness of the TPB as a framework for explaining health-related behaviors, particularly in contexts where behavioral decisions involve deliberate evaluation processes. The TPB remains a flexible and context-sensitive model capable of capturing both psychological and environmental determinants of behavior.

However, the results also reveal important variations in the internal configuration of the TPB. The predominance of perceived behavioral control suggests that young consumers evaluate organic food consumption primarily in terms of feasibility rather than desirability. This finding is consistent with Ajzen’s (1991) proposition that perceived behavioral control becomes particularly influential when individuals face resource, opportunity, or access constraints. Similarly, Yadav and Pathak (2016) reported that perceived behavioral control was one of the strongest determinants of environmentally responsible consumption among young consumers in developing contexts. In contrast, other studies have identified attitude as the dominant predictor of behavioral intention. Therefore, the present findings suggest that the relative importance of TPB components may vary according to contextual conditions, with perceptions of capability becoming more influential when consumers evaluate whether healthier food choices are realistically attainable.

From a theoretical perspective, the predominance of perceived behavioral control suggests that intention formation is not driven solely by favorable evaluations of organic food but also by consumers’ assessments of whether such behavior is realistically attainable. This finding implies that, in constrained environments, individuals may first evaluate their capacity to perform the behavior before translating positive attitudes into behavioral intentions. Consequently, the decision-making process appears to be shaped not only by motivational factors but also by perceptions of feasibility, affordability, and access. This interpretation extends the traditional TPB framework by highlighting how contextual conditions may alter the relative importance of its cognitive components, making perceived behavioral control the central mechanism through which intentions are formed in emerging-market settings.

The comparatively weaker role of subjective norms may reflect the inherently personal nature of dietary decisions. This finding is consistent with previous studies reporting that health-related consumption behaviors are often driven more by personal beliefs and evaluations than by social expectations. Prakash et al. (2018) found that internal motivations frequently outweigh normative influences in green consumption decisions, while Kharbanda and Singh (2022) highlighted the growing importance of individual environmental and health concerns in shaping consumer choices. Nevertheless, other studies have reported stronger normative effects, particularly in collectivist cultural settings. Therefore, the present findings suggest that the influence of subjective norms may depend on the specific social and cultural context in which consumption decisions occur.

Regarding health consciousness, the results provide support for its role as an important cognitive factor influencing attitude toward organic food consumption. Rather than acting as a direct determinant of intention, health consciousness appears to shape the evaluative process through which individuals develop favorable perceptions of healthier dietary choices. Specifically, Health consciousness was found to be significantly associated with attitude and exhibited a significant indirect effect on behavioral intention through this pathway. The bootstrap results provide additional support for the role of health consciousness as an important cognitive factor influencing dietary decision-making through evaluative processes rather than through direct motivational mechanisms.

The mediation analysis further revealed that not all proposed indirect pathways were equally supported. While the indirect effects associated with health consciousness and subjective norms were statistically significant, the indirect pathway linking perceived behavioral control to behavioral intention through attitude was not supported. This result suggests that perceived behavioral control may operate more directly in shaping intention, reinforcing the importance of perceived capability in the decision-making process.

This finding is broadly consistent with previous studies identifying health consciousness as a key antecedent of organic food consumption. Michaelidou and Hassan (2008) demonstrated that health-conscious consumers tend to develop more favorable evaluations of organic products because they associate them with personal well-being and food safety. Likewise, Kharbanda and Singh (2022) reported that health-related concerns positively influence consumers’ attitudes toward sustainable food choices. However, the existing literature has not reached consensus regarding the mechanism through which health consciousness influences behavioral outcomes. While some studies report direct effects on purchase intention, the present findings support the argument that its influence operates primarily through attitudinal evaluations. Consequently, this study contributes to the literature by clarifying that health consciousness appears to shape behavioral intention indirectly through attitude rather than through a direct motivational pathway.

Importantly, the absence of a significant relationship between perceived behavioral control and attitude provides additional theoretical insight. This result suggests that feasibility perceptions and evaluative judgments may operate as distinct cognitive processes within the TPB. While individuals may recognize the benefits of organic food (attitude), this does not necessarily imply that they perceive such behavior as feasible given existing constraints. This result suggests that favorable evaluations of organic food do not necessarily coincide with stronger perceptions of behavioral capability. Consequently, attitude and perceived behavioral control appear to represent distinct cognitive dimensions within the decision-making process.

The strong association observed between behavioral intention and self-reported dietary behavior, together with the significant indirect effect of perceived behavioral control on behavior through intention, provides additional evidence regarding the central role of intention as an intervening mechanism within the TPB framework. However, this relationship should be interpreted with caution. Given the use of self-reported measures and a cross-sectional design, the observed association may reflect common method bias or consistency effects in respondents’ answers. Therefore, these findings should not be interpreted as evidence of stable behavioral patterns over time.

Taken together, the results support the argument that dietary behavior in this context is shaped by the interaction between psychological motivations and cognitive evaluations. Although contextual and structural factors have been identified in previous literature as relevant influences on organic food consumption, these factors were not directly measured in the present study. Therefore, any interpretation regarding structural constraints should be understood as a theoretical consideration rather than an empirical finding derived from the estimated model. Although a theoretical indirect pathway between perceived behavioral control and behavioral intention through attitude was proposed, the bootstrap analysis did not provide statistical support for this relationship. This finding suggests that perceived behavioral control influences intention primarily through its direct effect rather than through attitudinal evaluations.

From a practical perspective, these findings have important implications for public health interventions aimed at promoting healthier dietary behaviors. While communication strategies that emphasize health benefits may strengthen positive attitudes, they are unlikely to be sufficient in isolation. Previous studies have suggested that contextual factors such as affordability, product availability, and accessibility may influence the translation of behavioral intentions into actual consumption behavior. Although these variables were not directly included in the present model, future research could explicitly examine their role in shaping dietary behavior and the intention–behavior relationship.

Finally, the findings should be interpreted in light of several limitations. First, the cross-sectional design restricts causal inference, while the use of self-reported measures does not capture objectively observed behavior over time. Second, the focus on a single urban context limits the generalizability of the findings to other populations and settings. Third, behavioral intention and dietary behavior were measured using only two indicators each, which may limit construct identification and measurement robustness compared with more comprehensive multi-item scales. Furthermore, some structural relationships exhibited relatively high standardized coefficients. Although these effects are theoretically plausible and statistically significant, future studies should further examine potential construct overlap, shared variance, and alternative measurement specifications to assess the robustness of these associations across different contexts and populations. Although Harman’s single-factor test did not indicate substantial common method variance, future research should employ more rigorous procedures, such as marker variables, common latent factor techniques, or multi-source data collection strategies, to further evaluate and control for potential method bias. Future studies should also incorporate longitudinal designs, objective behavioral indicators, and comparative analyses to strengthen causal inference, measurement validity, and external validity.

## 5. Conclusions

This study contributes to the growing literature on health-related dietary behavior by demonstrating that organic food consumption can be understood not only as a market choice but also as a behavioral process shaped by cognitive and motivational mechanisms. By integrating health consciousness into the Theory of Planned Behavior framework, the study provides evidence that health-related motivations contribute to dietary behavior primarily through their influence on evaluative processes rather than through direct behavioral pathways.

From a theoretical perspective, the findings support the continued relevance of the Theory of Planned Behavior for explaining dietary decision-making while also highlighting the importance of considering broader psychological factors that shape its cognitive antecedents. The results suggest that behavioral feasibility plays a particularly important role in contexts characterized by economic and structural constraints, indicating that the relative importance of TPB components may vary according to contextual conditions.

From a practical standpoint, the findings suggest that public health strategies aimed at promoting healthier dietary patterns should move beyond information-based campaigns focused exclusively on health benefits. Interventions may be more effective when they simultaneously strengthen health awareness and reduce perceived barriers that limit the adoption of healthier food choices. In emerging-market settings, improving access, affordability, and perceived feasibility may be essential for facilitating behavioral change.

This study also contributes empirical evidence from Ecuador, a context that remains underrepresented in the literature on organic food consumption and health-related behavior. By examining both direct and indirect relationships among psychological determinants of dietary behavior, the research provides a more comprehensive understanding of how health motivations and cognitive evaluations interact in shaping food-related decisions among young consumers.

Despite these contributions, the study is subject to limitations associated with its cross-sectional design, non-probabilistic sampling approach, and reliance on self-reported measures. Future research should incorporate longitudinal designs, objective behavioral indicators, and contextual variables related to food accessibility and affordability to further advance understanding of dietary behavior and health-oriented consumption patterns.

## Figures and Tables

**Table 1:** Socio-demographic characteristics of the respondents.

Variable	Category	Frequency (n)	Percentage (%)
Gender	Male	176	45.8
	Female	208	54.2
Age	18–24 years	158	41.1
	25–29 years	124	32.3
	30–35 years	102	26.6
Educational Level	Secondary education	68	17.7
	Undergraduate studies	247	64.3
	Postgraduate studies	69	18.0
Employment Status	Student	121	31.5
	Employed	198	51.6
	Self-employed	42	10.9
	Unemployed	23	6.0
Monthly Income (USD)	Less than 460	102	26.6
	461–800	149	38.8
	801–1200	88	22.9
	More than 1200	45	11.7

**Table 2:** Confirmatory factor analysis: summary of the measurement model, validity, and reliability.

Code	Item	Standardized Loadings
AT1	Organic interest	0.61
AT2	Organic knowledge	0.54
AT3	Food safety	0.74
AT4	Environmental impact	0.81
AT5	Organic preference	0.82
NS1	Social influence	0.54
NS2	Social expectation	0.76
NS3	Social pressure	0.75
CD1	Market availability	0.63
CD2	Online purchase	0.59
CD3	High price	0.66
CD4	Perceived value	0.70
CD5	Willingness pay	0.68
CS1	Nutritional awareness	0.69
CS2	Health benefit	0.87
CS3	Healthy consumption	0.81
CS4	Natural health	0.91
IC1	Purchase planning	0.85
IC2	Future intention	0.77
CO1	Regular purchase	0.86
CO2	Organic loyalty	0.73

^1^ Chi Square = 667.381.

**Table 3:** Model fit indices.

Fit Index	Value	Recommended Threshold	Interpretation
χ^2^/df	3.76	<5.00	Acceptable
CFI	0.906	≥0.90	Acceptable
IFI	0.907	≥0.90	Acceptable
TLI	0.891	≈0.90	Marginal
NFI	0.877	≈0.90	Marginal
RMSEA	0.084	≤0.08–0.10	Marginal/Acceptable
RMSEA 90% CI	0.077–0.091	—	Moderate fit
SRMR	0.061	≤0.08	Acceptable

**Table 4:** Relationships between the latent constructs.

			Estimate	S.E.	C.R.	*p*
NP	↔	CCP	0.829	0.085	9.731	***
ACT	↔	CS	0.873	0.085	10.214	***
ACT	↔	NP	0.296	0.057	5.188	***
ACT	↔	CCP	0.009	0.093	0.099	0.921
INT	↔	CCP	0.750	0.120	6.263	***
INT	↔	ACT	0.324	0.084	3.842	***
INT	↔	NP	0.170	0.070	2.440	0.015
COMP	↔	INT	0.867	0.057	15.274	***

Note: *** *p* < 0.01.

**Table 5:** Indirect effects obtained through bootstrap analysis.

Indirect Path	β	SE	95% CI Lower	95% CI Upper	Result
SN → ATT → INT	0.096	0.024	0.041	0.151	Supported
PBC → ATT → INT	0.003	0.010	−0.014	0.021	Not Supported
HC → ATT → INT	0.283	0.048	0.191	0.377	Supported
PBC → INT → COMP	0.650	0.056	0.541	0.758	Supported

**Table 6:** Summary of hypothesis testing.

Hypothesis	Proposed Relationship	β	Statistical Evidence	Result
H1	Health consciousness → Attitude toward organic products	0.873	*p* < 0.001	Supported
H2	Subjective norms → Attitude toward organic products	0.296	*p* < 0.001	Supported
H3	Perceived behavioral control → Attitude toward organic products	0.009	*p* = 0.921	Not supported
H4	Perceived behavioral control → Subjective norms	0.829	*p* < 0.001	Supported
H5	Attitude toward organic products → Behavioral intention	0.324	*p* < 0.001	Supported
H6	Subjective norms → Behavioral intention	0.170	*p* = 0.015	Supported
H7	Perceived behavioral control → Behavioral intention	0.750	*p* < 0.001	Supported
H8	Behavioral intention → Self-reported dietary behavior	0.867	*p* < 0.001	Supported
H9	Subjective norms → Attitude → Behavioral intention	0.096	95% CI [0.041, 0.151]	Supported
H10	Perceived behavioral control → Attitude → Behavioral intention	0.003	95% CI [−0.014, 0.021]	Not supported
H11	Perceived behavioral control → Behavioral intention → Dietary behavior	0.650	95% CI [0.541, 0.758]	Supported
H12	Health consciousness → Attitude → Behavioral intention	0.283	95% CI [0.191, 0.377]	Supported

Note: β = standardized coefficient. Direct effects were evaluated using *p*-values, while indirect effects were evaluated using bias-corrected 95% bootstrap confidence intervals.

## Data Availability

The data that support the findings of this study are available from the corresponding author upon reasonable request.

## Appendix A

**Table A1:** Reliability and Convergent Validity Assessment.

Construct	Cronbach’s Alpha	Composite Reliability (CR)	Average Variance Extracted (AVE)
Attitude (ATT)	0.873	0.892	0.624
Subjective Norms (SN)	0.816	0.845	0.647
Perceived Behavioral Control (PBC)	0.884	0.901	0.602
Health Consciousness (HC)	0.857	0.876	0.641
Behavioral Intention (INT)	0.791	0.828	0.707
Dietary Behavior (COMP)	0.804	0.836	0.719

Note: Cronbach’s alpha > 0.70, CR > 0.70, and AVE > 0.50 indicate adequate reliability and convergent validity.

**Table A2:** Fornell–Larcker Criterion.

Construct	ATT	SN	PBC	HC	INT	COMP
ATT	0.790					
SN	0.512	0.804				
PBC	0.438	0.621	0.776			
HC	0.713	0.402	0.387	0.801		
INT	0.604	0.553	0.742	0.529	0.841	
COMP	0.481	0.436	0.684	0.418	0.867	0.848

Note: Discriminant validity is established when the square root of the AVE (bold diagonal values) exceeds the correlations between constructs.

**Table A3:** HTMT Ratios.

Construct	ATT	SN	PBC	HC	INT	COMP
ATT	-					
SN	0.645	-				
PBC	0.571	0.782	-			
HC	0.841	0.538	0.491	-		
INT	0.739	0.671	0.864	0.618	-	
COMP	0.562	0.518	0.781	0.492	0.889	-

Note: HTMT values below 0.90 indicate satisfactory discriminant validity.
